# Responses of earliness and lateness genes for heading to different photoperiods, and specific response of a gene or a pair of genes to short day length in rice

**DOI:** 10.1186/s41065-019-0109-5

**Published:** 2019-12-18

**Authors:** Birendra Bahadur Rana, Misa Kamimukai, Mukunda Bhattarai, Yohei Koide, Masayuki Murai

**Affiliations:** 1 The United Graduate School of Agricultural Sciences, Ehime University, Matsuyama, Ehime Japan; 2 Nepal Agriculture Research Council (NARC), Kathmandu, Nepal; 3 Research Faculty of Agriculture, Hokkaido University, Sapporo, Japan; 4 Faculty of Agriculture and Marine Science, Kochi University, 200 Otsu, Monobe, Nankoku, Kochi 783-0093 Japan

**Keywords:** Heading time, Day length, Earliness genes, Lateness genes, Photo-sensitivity, Basic vegetative growth, Rice

## Abstract

**Background:**

Heading time is an important trait for regional and seasonal adaptabilities in rice, and is controlled by genetic factors in relation with environmental factors, mainly day length and temperature. The following genes controlling heading were examined for their responses to six different environmental conditions involving different day lengths using five early near-isogenic lines (NILs) of T65-R and three late NILs of T65wx: two earliness genes, *Ef1* and *Efx* controlling basic vegetative phase (BVG), and *m-Ef1*, the enhancer to the former gene; and two lateness genes, *Se1-pat*(t) and *se-pat* controlling photo-sensitivity and BVG, respectively. T65-R and T65-T were different accessions of Taichung 65. T65wx is a NIL of T65-T carrying *wx*.

**Results:**

The five early NILs of T65-R were in the order of ER50 (*Ef1*, *Efx*, *m-Ef1*) < ER40 (*Ef1*, *m-Ef1*) ≤ ER20 (*Ef1*, *Efx*) < ER1 (*Ef1*) ≤ ER21 (*Efx*) < T65-R regarding days to heading (DTH) under two spring-sowing and one summer-sowing paddy field (PF) conditions. The three late NILs of T65wx were in the order of LF3 (*Se1-pat*(t)) ≤ LF2 (*Se1-pat*(t), *se-pat*) ≤ T65wx < LF1 (*se-pat*) under two short-day conditions (10-h photoperiod condition with artificial-light and natural short-day condition from autumn to winter). The NILs and T65wx were in the order of T65wx < LF3 < LF1 < LF2 under the two spring-sowing PF (long day) conditions. T65-R (*Ac-ef1*) was 2.8 or 5.1 days earlier in DTH than T65-T (*ac-ef1*) under the two spring-sowing PF conditions. However, T65-R was 19 and 10 days earlier than T65-T under the two short-day conditions.

**Conclusions:**

Earliness gene(s) and their combinations reduced DTH regardless of photoperiod lengths. *Se1-pat*(t) increased DTH under long-day conditions but decreased it under short-day conditions, while *se-pat* elongated DTH under both short-day and long-day conditions indicating that *se-pat* is responsible for BVG. The *se-pat* increased DTH by adding its effect over that of *Se1-pat*(t) under long-day conditions. However, this increasing effect of DTH by *se-pat* was almost completely masked when it coexisted with *Se1-pat*(t) under the short-day conditions. Notably, the response of *Ac-ef1* to day length was found to delay heading under the short-day conditions.

## Introduction

Heading time is an important ecological characteristic in rice, and is related to regional and seasonal adaptabilities [2, 21, 40]. It is controlled by genetic factors in relation with environmental factors, mainly day length and temperature. According to a number of genetical studies, many genes and/or alleles control photoperiod sensitivity and length of basic vegetative growth (“BVG”) in rice.

The growing period of rice from sowing to maturity is divided into the vegetative and reproductive phases [2, 15, 39]. The vegetative phase is further divided into the BVG and photoperiod-sensitive phases. The reproductive phase is classified into the period from differentiation of panicle primordium to heading and that from heading to maturity. The duration from appearance of panicle primordium to heading is about 30 days in *japonica* rice varieties ordinarily [1, 30]. Therefore, heading time is principally determined by both the BVG and photo-sensitive phases. The BVG phase is the minimum duration of vegetative growth from sowing to initiation of panicle development under the condition of short day (“SD”) and higher temperature. The photoperiod-sensitive phase can be estimated by subtracting the period of BVG from the growth duration from sowing to heading under a long-day (“LD”) condition [2]. Varieties of Hokkaido in Japan, one of the northernmost rice growing regions in the world, possess weak or no photo-sensitivity [8, 15], because photosensitive varieties are delayed in heading due to LD conditions in summer, and cannot mature sufficiently before the first frost in late autumn. According to Okumoto et al. [21], a lateness gene controlling photo-sensitivity, *E1*, is commonly harbored in early, middle, and late heading varieties grown in south-western region of Japan, and another photo-sensitivity gene, *E2*, and/or other genes are related to the variation in heading time.

Shigetoshi Sato (University of the Ryukyus), developed five near-isogenic lines (NILs) of an accession of Taichung 65 maintained at the university (“T65-R”), which harbor either or both of earliness genes *Ef1* and *Efx* in all of the five NILs, and the enhancer to the former gene, *m-Ef1*, in two of the lines ([9, 24, 25], Table 1 and Table 2). *Ef1* is a dominant allele for earliness at the *Ef1* locus controlling the BVG on chromosome 10 that accelerates heading by 7 to 13 days [31–35, 37]. Taichung 65 harbors its recessive allele *ef1* at the locus which increases the BVG period. *Ef1* and other dominant alleles are widely distributed in not only early-heading varieties of Japan and a representative middle-heading variety of warmer regions of Japan, ‘Nipponbare’, but also Chinese and tropical *indica* varieties [23, 24, 32, 37]. On the other hand, *ef1* is a specific allele found only in some Taiwanese *japonica* varieties [23]. *Efx* is a dominant allele for earliness at a locus on chromosome 3 [24, 28]: its effect of reducing BVG was accelerated by higher temperature, and this accelerating effect was masked by *Ef1* [24]. A recessive allele, *m-Ef1*, is the enhancer for *Ef1* [37], which is identical with *e1* allele at the *E1* locus on chromosome 7 [22], and *E1* (= *Hd4*) is a dominant allele for photo-sensitivity over *e1* for non-photo-sensitivity [8, 17, 40].

**Table 1:** Five early NILs and T65-R, T65-T, and three late NILs and T65wx used in the present study

Line/ Variety	Donor			Recurrent parent	No. of backcrosses	References
	Variety	Sub- species	Origin			
ER50^a^	A58	*japonica*	Japan	ER1	7	Sato unpublished, Tsai [35], and Itoh et al. [9]
ER40^a^	A58	*japonica*	Japan	ER1	8	Sato et al. [24], Tsai [35], and Itoh et al. [9]
ER20	A58	*japonica*	Japan	T65-R	9	Sato et al. [24], and Itoh et al. [9]
ER1	A58	*japonica*	Japan	T65-R	10	Sato et al. [24, 25], and Itoh et al. [9]
ER21	A58	*japonica*	Japan	T65-R	8	Sato et al. [24], and Itoh et al. [9]
T65-R^b^						Tsai [36], and Sato et al. [24]
T65-T^c^						Tsai and Oka [31], and Tsai [36]
T65wx	Kinoshita-mochi	*japonica*	Japan	T65 T	8	Personal communication from Yoshio Sano (Hokkaido University)
LF3	Patpaku	*indica*	India	T65wx	7	Dung et al. [4], and Itoh et al. [9]
LF1	Patpaku	*indica*	India	T65wx	7	Dung et al. [4], and Itoh et al. [9]
LF2	Patpaku	*indica*	India	T65wx	7	Dung et al. [4], and Itoh et al. [9]

^a^*m-Ef1*^*b*^ was donated from Bozu 5

^b^Accession of Taichung 65 maintained at The University of the Ryukus, Okinawa, Japan

^c^Accession of Taichung 65 maintained at National Chung Hsing University, Taichung, Taiwan

**Table 2:** Genotypes of T65-R, T65wx and their early and late NILs

Line/ Variety	Genes/alleles					
	*Ac-ef1*	*Ef1*	*Efx*	*m-Ef1*^*b* a^	*Se1-pat*(t)	*se-pat*
ER50	*Ac-ef1*	*Ef1*^*A58*^	*Efx*	*m-Ef1*^*b*^	*se1*	*se-pat*^*+*^
ER40	*Ac-ef1*	*Ef1*^*A58*^	*efx*	*m-Ef1*^*b*^	*se1*	*se-pat*^*+*^
ER20	*Ac-ef1*	*Ef1*^*A58*^	*Efx*	*M-Ef1*	*se1*	*se-pat*^*+*^
ER1	*Ac-ef1*	*Ef1*^*A58*^	*efx*	*M-Ef1*	*se1*	*se-pat*^*+*^
ER21	*Ac-ef1*	*ef1*	*Efx*	*M-Ef1*	*se1*	*se-pat*^*+*^
T65-R	*Ac-ef1*	*ef1*	*efx*	*M-Ef1*	*se1*	*se-pat*^*+*^
T65-T	*ac-ef1*	*ef1*	*efx*	*M-Ef1*	*se1*	*se-pat*^*+*^
T65wx	*ac-ef1*	*ef1*	*efx*	*M-Ef1*	*se1*	*se-pat*^*+*^
LF3	*ac-ef1*	*ef1*	*efx*	*M-Ef1*	*Se1-pat*(t)	*se-pat*^*+*^
LF1	*ac-ef1*	*ef1*	*efx*	*M-Ef1*	*se1*	*se-pat*
LF2	*ac-ef1*	*ef1*	*efx*	*M-Ef1*	*Se1-pat*(t)	*se-pat*

^a^*m-Ef1*^*b*^ was donated from Bozu 5

In addition to *E1*, several major genes controlling photoperiod sensitivity, e.g. *Se1* and *Hd2* have been reported [4, 8, 18, 40, 41, 46]. Furthermore, the three combined pairs of two different alleles at two of the *Se1*, *E1* and *Hd2* loci exhibited additive effects and epistatic interactions between them in heading [16, 26]. The *Se1* locus on chromosome 6 involves at least three alleles: the magnitudes of delaying heading are in the order of *Se1-u* > *Se1-n* > *Sel-e*, where the latter allele is non-functional for photo-sensitivity [43, 45]. *Se1-e* is distributed in rice varieties grown in Hokkaido prefecture and the Tohoku district of Japan [7, 46], while *Se1-n* is harbored in middle- and late-heading varieties grown in warmer regions of Japan [46]. *Se1-u* is harbored in an *indica* variety ‘Morak Sepilai’ [43]. Yano et al. [42] succeeded in the cloning of the *Hd1* locus, identical with the *Se1* locus, and indicated that the *hd1* allele harbored by ‘Kasalath’ is non-functional and the *Hd1* allele of ‘Nipponbare’ is functional. The *Hd1* locus involves a high level of polymorphisms in diverse germplasm of rice involving *indica* and *japonica* [29]. According to Ebana et al. [5], functional allele(s) at the *Hd1* locus are harbored in *japonica* and *indica* varieties originating from China and tropical countries.

Sano [4] developed three NILs of T65wx carrying either or both of *Se1-pat*(t) and *se-pat*, the common donor of which was an *indica* variety, ‘Patpaku’. *Se1-pat*(t) is a dominant allele at the *Se1* locus [4]. T65wx is a NIL carrying *wx* (glutinous) allele of the accession Taichung 65 maintained at National Chung Hsing University of Taiwan (“T65-T”) [35, 36]. The *se-pat* locus is located between the *C* (Chromogen) and *wx* loci, and is distant from the *Se1* locus on the same chromosome 6 [3]. Results obtained by Hagiwara et al. [6] suggest that *se-pat* elongates the BVG, even though they did not clearly conclude such. The three late NILs were used in the present study.

Tsai [35] reported that T65-R headed 7.7 and 4.2 days earlier, respectively, than T65-T in the first and second crop seasons in Taichung, Taiwan, and assumed that these differences were caused by *Ac-ef1* in T65-R which accelerated heading by affecting the character expression of *ef1*, and another genetical factor.

The five early NILs, T65-R, T65-T, T65wx, and the three late NILs were grown under six different environmental conditions: SD (10-h photoperiod) and 13.5-h photoperiod conditions in an artificial light-type (abbreviated as “Art-L”) growth chamber (hour is abbreviated as “h”) and a natural light-type (abbreviated as “Nat-L”) growth chamber from autumn to winter (SD condition), and two spring-sowing paddy-field (abbreviated as “PF”) conditions and a summer-sowing PF condition. From the data obtained, influences of day length on the character expressions of the earliness and lateness genes and some combinations of them were examined as follows: 1) whether character expressions of *Ef1*, *Efx* and *m-Ef1* (= *e1*) are affected by day length or not, 2) whether character expression of *Ac-ef1* is influenced under different photoperiod conditions or not, 3) whether there are any genic interactions between *Se1-pat*(t) and *se-pat* under the different photoperiod conditions or not. In addition, it could be ascertained that identical results are obtained under both artificial-light and natural-light SD (or LD) conditions. From the results obtained, an exceptional and interesting reaction of *Ac-ef1* was detected: it delayed heading under the two SD conditions. An almost complete suppression of the effect of *se-pat* on increasing number of days to heading was observed with the co-existence of *Se1-pat*(t) under the SD conditions.

## Materials and methods

### Plant materials

Tables 1 and 2 show the five early NILs of T65-R and the three late NILs of T65wx used in the present experiments, all of which were donated by Yoshio Sano (Hokkaido University). The common donor of *Ef1* and *Efx* for the corresponding early NILs of T65-R was A58 (Kokushokuto-2), a native variety of Hokkaido. The donor of *m-Ef1*^*b*^ for ER50 and ER40 was Bozu 5, a *japonica* variety of Hokkaido. For the five early NILs, the number of backcrosses with T65-R or ER1 was 7 to 10 (Table 1). As shown in Table 2, the alleles mentioned above harbored by the five NILs were as follows: *Ef1* and *Efx* in ER1 and ER21, respectively; both of them in ER20; *Ef1* and *m-Ef1*^*b*^ in ER40; all of the three alleles in ER50.

LF3, LF1 and LF2 harbored *Se1-pat*(t), *se-pat* and both of them, respectively, the common donor of which was ‘Patpaku’ (Tables 1 and 2). The recurrent parent T65wx is a NIL carrying *wx* originating from a *japonica* variety, ‘Kinoshita-mochi’, being developed after 8 backcrosses with T65-T. The number of backcrosses with T65wx was 7 in each of the three late NILs.

### Cultivations in paddy field

Sterilized seeds were sown into plastic trays filled with a granulated soil containing N, P_2_O_5_ and K_2_O and adjusted at pH 4.5, on 2nd May 2011, 14th July 2014 and 24th April 2018, and were grown in the Nat-L growth chamber. Twelve- or thirteen-day-old seedlings were transplanted, at a spacing of 30 × 15 cm (22.2 hills/m^2^) with a single seedling per hill, to an experimental field at the Faculty of Agriculture (present name: the Faculty of Agriculture and Marine Science), Kochi University, Nankoku (33°33′N). The total amount of fertilizers applied by basal and topdressings were at the N levels of 10.0, 7.9 and 4.5 g/m^2^ in 2011, 2014 and 2018, respectively, together with adequate amounts of P_2_O_5_ and K_2_O.

According to Kuriyama [15], and Yokoo and Kikuchi [44], the critical time is 35 days before heading, at which photosensitive varieties prepare to initiate panicle development in response to SD length. Day lengths at 35 days before heading in the material lines in 2011, 2013, 2014 and 2018 were taken from the database of the National Astronomical Observatory of Japan (http://eco.mtk.nao.ac.jp/cgi-bin/koyomi/koyomix.cgi) [19], as shown in Table 3. The monthly average temperatures from May to October in 2011, 2014 and 2018 were taken from a database of the Japan Meteorological Agency (http://www.jma.go.jp/jma/menu/report.html) [10], as shown in Table 4.

**Table 3:** Day lengths on 35 days before heading in T65-R, T65wx and their early and late NILs, and T65-T

Line/ Variety	Paddy field			Growth chamber^c^
	2011^a^	2018^a^	2014^b^	2013–2014
ER50^d^	14:13^f^	14:10^f^	13:44^f^	10:41^f^
ER40^d^	14:19	14:17	13:33	10:31
ER20^d^	14:19	14:17	13:25	10:43
ER1^d^	14:22	14:20	13:20	10:28
ER21^d^	14:23	14:21	13:10	10:13
T65-R	14:23	14:23	13:04	9:59
T65-T	14:22	14:23	13:07	10:07
T65wx	14:21	14:23	13:04	10:06
LF3^e^	13:45	13:34	12:59	10:26
LF1^e^	13:35	13:18	12:28	9:56
LF2^e^	13:10	12:57	13:02	10:24

Source: National Astronomical Observatory of Japan (http://eco.mtk.nao.ac.jp/cgi-bin/koyomi/koyomix.cgi)

^a^Spring sowing

^b^Summer sowing

^c^Natural light-type

^d^Early NILs of T65-R

^e^Late NILs of T65wx

^f^Hours: minutes

**Table 4:** Monthly average temperatures (°C) during the growth of the material lines after transplanting in the three experimental years in the paddy field

Year	May	June	July	August	September	October
2011	19.1	22.8	26.0	27.1	24.6	19.5
2018	19.6	23.0	27.3	27.9	24.2	18.9
2014	– ^a^	– ^a^	26.0	26.2	23.7	20.1

Source: Japan Meteorological Agency (http://www.jma.go.jp/jma/menu/report.html). Site of observation: Nankoku-Nissho meteorological-observation point, near the experimental paddy field

^a^Before the material lines were transplanted

The date of first heading in each plant was recorded. The culm length and panicle length of the highest culm in each plant were measured for 19 to 24 plants per line in 2018.

### Pot culture

Experiments in the Art-L and Nat-L growth chambers were performed by pot culture. About 350 g of the granulated soil mentioned above was taken into cylindrical pots with an inside diameter and depth of 15.6 and 19.0 cm, respectively. A slow release fertilizer ECOLONG® 424–180 type (3% of each nutrient element is readily available) manufactured by JCAM AGRI Co. Ltd., was applied to each pot at the rate of 0.54 g each of N, P_2_O_5_ and K_2_O. Sterilized seeds were soaked at about 27 °C for 2 days. These 2 days were added to the days to heading (abbreviated as “DTH”) for every line/variety in the pot-culture experiments mentioned below. Six soaked seeds were sown circularly along the wall of each pot and were grown in the growth chambers, together with some extra seeds sown at the center of each pot which were removed after replacing non-germinated seeds and weak seedlings at regular sites at about the three-leaf stage. Three to seven pots per line/variety were used in each of the environmental conditions. The sowing date in the Nat-L growth chamber was 11th October 2013. The Art-L growth chambers used in the experiments were illuminated at about 600 μ mol/m^2^/s at 60 cm from the floor, using a combination of florescent lamps and electric bulbs. Uwatoko et al. [38] suggested that the photo-sensitivities of various genotypes by combining *Se1-pat*(t), *E1*, *Ef1* and their recessive alleles were appropriately evaluated by photoperiod treatments at not only 28 °C but also 22 °C. In the present study, temperature in both types of the growth chambers was set at 23 °C throughout the experiments. This rather low temperature was used to avoid excessive spindly-growth under the low light intensity in the Art-L growth chamber. Tillers were removed once or twice during the growth period. The first heading date of each plant was recorded.

The number of developed leaves in each plant was recorded for 5 to 7 and 11 to 19 plants per line, respectively, under 10-h and 13.5-h photoperiod conditions in the Art-L growth chambers.

### Definition of short and long photoperiods

Kuriyama [15] suggested that photosensitive rice varieties originating from Japan, India, Myanmar and Indonesia had the minimum number of DTH under a condition of 9-h, 10-h or 11-h photoperiod, and DTH increased from a 12-h condition and thereafter in the varieties except a Japanese one ‘Zuiho’. Uwatoko et al. [38] obtained similar results by using the eight early and late NILs of T65wx combined with *Se1-pat*(t), *Ef1*, *E1* and their respective recessive alleles. Furthermore, highly photo-sensitive varieties originating from tropical and sub-tropical Asian countries failed to attain heading even under a 12.5-h photoperiod condition; however, ‘Zuiho’ normally headed under a 13-h photoperiod condition, and attained heading under a 14-h photoperiod condition despite delay in heading of about one month [15]. Accordingly, we consider photoperiods longer than 14 h inclusive as LD, and those shorter than 11 h inclusive as SD. The *Se1-pat*(t) NIL of T65wx, almost the same as LF3, did not attain heading under a 14-h photoperiod conditions at both 22 °C and 28 °C [38]. A similar result was reported by Dung et al. [4]. Results obtained by Uwatoko et al. [38] suggest that the photo-sensitivity of *Se1-pat*(t) was able to be evaluated under 13.5-h photoperiod conditions at the two temperatures. Accordingly, we used a 13.5-h photoperiod at 23 °C to evaluate the photo-sensitivities of LF3 and the other lines by means of DTH.

## Results

### Day lengths at the critical stage for preparing panicle development under the PF and Nat-L chamber conditions, and monthly average temperatures in the experimental years in the PF

Table 3 shows day lengths at 35 days before heading in the early NILs, T65-R, T65-T, T65wx and the late NILs which were calculated from daily sunrise and sunset times in the three experimental years in the PF, and autumn to winter in 2013–2014 in the Nat-L growth chamber, being taken from the database of National Astronomical Observatory of Japan. Under the PF conditions in 2011 and 2018, day lengths at the critical stage (35 days before heading) in the five early NILs, T65-R, T65-T and T65wx were from 14:13 (hours: minutes) to 14:23 and 14:10 to 14:23 in 2011 and 2018, respectively, suggesting long-day conditions on their respective days at the critical stage in both years. It seems that their photo-sensitivities are small or not so large to prevent panicle initiation under the long day conditions in summer. Under the summer-sowing PF condition in 2014, the day lengths were from 13:04 to 13:44 in the eight lines-varieties, suggesting that those at the critical stage seem to have not been typically long but not short.

For the *Se1-pat*(t)-carrying lines LF3 and LF2, day lengths at the critical stage were 13:45 and 13:10, and 13:34 and 12:57, respectively, in 2011 and 2018 (Table 3). Those were 12:59 and 13:02, respectively, in LF3 and LF2, in 2014. In this year, the two NILs headed in October (see DTH in Table 5 and the sowing date of 14th July 2014 in the materials and methods), indicating their panicle primordia began to develop in September during which the monthly average temperature was not low (23.7 °C). Therefore, their panicle developments may have been prepared to initiate in day lengths shorter than their threshold day lengths in the three experimental years.

**Table 5:** DTHs of T65-R, T65wx and their early and late NILs under the six environmental conditions

Line/ Variety	Spring- sowing paddy field in 2011^a^	Spring-sowing paddy field in 2018^b^	Summer-sowing paddy field in 2014^c^	13.5-h photo-period^d, e^	10-h photo-period^d, f^	Natural light-type growth chamber^g^
ER50	65.0 a	69.2 a	56.9 a	67.8 a	65.0 a	61.9 a
ER40	72.9 b	77.1 b	63.4 b	82.6 bc	73.2 b	67.6 b
ER20	72.9 b	78.1 b	67.8 c	70.2 a	67.3 a	60.7 a
ER1	78.5 c	81.5 c	70.3 d	85.4 c	74.3 b	69.5 b
ER21	81.3 d	83.9 d	75.1 e	77.7 b	85.3 c	80.9 c
T65-R	88.8 e	88.2 e	78.1 f	107.2 d	114.4 g	95.3 e
T65-T	93.9 f	91.0 f	77.2 f	107.2 d	95.1 f	85.4 cd
T65wx	96.1 g	92.2 f	78.4 f	113.2 e	92.1 e	86.8 d
LF3	129.3 h	143.8 g	81.4 g	137.3 g	86.8 cd	70.5 b
LF1	135.0 i	152.8 h	96.1 h	130.4 f	125.9 h	111.5 f
LF2	148.0 j	163.6 i	79.1 f	145.3 h	90.7 de	71.6 b

Values followed by the same letter within the same column are not statistically different at the 5% level, in accordance with Tukey-Kramer’s method (Sokal and Rohlf [27])

^a^27 plants per line were measured

^b^23 to 27 plants per line were measured

^c^9 to 16 plants per line were measured

^d^Artificial light-type growth chamber

^e^11 to 17 plants per line were measured

^f^15 to 40 plants per line were measured

^g^9 to 17 plants per line were measured

Day lengths at the critical stage in LF1 were 13:35, 13:18 and 12:28, respectively, in 2011, 2018 and 2014, suggesting that day lengths at the critical stage seem to have been intermediate between LD and SD.

Under the Nat-L chamber condition, the day lengths were from 9:56 to 10:41 for all of the 11 lines, indicating that their panicle developments were prepared to initiate under the SD condition (Table 3).

As shown in Table 4, the monthly average temperature increased from May to August, and decreased from August to October in 2011 and 2018, like in ordinary years. In 2014, the monthly average temperatures were 26.0 °C in July, 26.2 °C in August, and 23.7 °C in September, suggesting that the temperature was high or rather high during the vegetative growths of the 11 lines, because LF1 headed on 18th October at the latest (its panicle development initiated on about 18th September) (Table 5).

### DTHs of T65-R and its early NILs under the six environmental conditions involving various photoperiod lengths

Under the spring-sowing PF condition in 2011, the DTH of T65-R was 88.8 days (Table 5). The DTH of ER50 was 23.8 days shorter than that of T65-R. Both ER40 and ER20 were 19.9 days earlier in heading than T65-R, and ER1 and ER21 were 10.3 and 7.5 days earlier, respectively, than T65-R. The order of DTH in the early lines and T65-R in 2018 was identical with that in 2011, with one exception that ER40 = ER20 in 2011 while ER40 ≤ ER20 in 2018 (≤ indicates non-significant difference) (Table 5). The five early lines and T65-R were − 0.6 to 5.2 days (3.1 days in average) longer in DTH in 2018 than those in 2011, probably because the sowing day was 8 days earlier in 2018 than in 2011. The order of DTH in the lines and T65-R under the summer-sowing PF condition in 2014 was identical with that in 2018, although their DTHs under the former condition were 8.8 to 13.7 days shorter than under the latter condition. This result was caused by higher temperature after transplanting in 2014 than in 2018 (Table 4).

The order of DTH in ER50, ER40, ER1 and T65-R except ER20 and ER21 under the 13.5-h photoperiod condition in the Art-L growth chamber was the same as that in 2018; and the DTHs of ER20 and ER21 were shorter than those expected from their DTHs in the three PF conditions (Table 5). ER40 (*m-Ef1* = *el*, *Ef1*) was earlier in heading than ER1 (*M-Ef1* = *E1*, *Ef1*), and similarly, ER50 (*m-Ef1* = *e1*, *Ef1*, *Efx*) was earlier than ER20 (*M-Ef1* = *E1*, *Ef1*, *Efx*), although being not significant statistically at the 5% level in both cases.

The order of DTH in T65-R and all of the lines except ER20 under the 10-h photoperiod condition in the Art-L growth chamber was the same as that in 2018, although the DTH of ER20 was shorter than that expected from its DTHs in the three PF conditions (Table 5). The lines and T65-R were in the order of ER20 ≤ ER50 < ER40 ≤ ER1 < ER21 < T65-R under the Nat-L growth chamber condition in which the day lengths at the critical stage were short (< indicates significant differences); this order is similar to that in the 10-h photoperiod condition. The DTHs of the five early NILs were 3.1 to 6.6 days shorter in the latter condition than in the former condition. Moreover, that of T65-R was 19.1 days shorter in the former condition than in the latter condition. It is noteworthy that under each SD condition, ER40 (*m-Ef1* = *e1*, *Ef1*) was not significantly different from ER1 (*M-Ef1* = *E1*, *Ef1*), and ER50 (*m-Ef1* = *e1*, *Ef1*, *Efx*) was not significantly different from ER20 (*M-Ef1* = *E1*, *Ef1*, *Efx*) (Table 2 and Table 5), which were different from the results regarding the four lines under the two spring-sowing PF (LD) conditions.

### Comparison of DTH between T65-R and T65-T under the six environmental conditions involving various day lengths

T65-R was 5.1 and 2.8 days earlier, respectively, in heading than T65-T under the spring-sowing PF conditions in 2011 and 2018 (Table 5). Day lengths at the critical stage for T65-R and T65-T were 14:22 or 14:23 in both years indicating LD conditions (Table 3). T65-R was 0.9 day later in heading than T65-T under the summer-sowing PF condition, although being not statistically significant, in which day lengths at the critical stage were 13:04 and 13:07, respectively. T65-R was identical with T65-T in DTH under the 13.5-h photoperiod condition. However, under the 10-h photoperiod condition, T65-R was 19.3 days later than T65-T. Under the Nat-L growth chamber condition, T65-R was 9.9 days later than T65-T, in which day lengths at their critical stage were 9:59 and 10:07, respectively, for the former and the latter accessions. Hence, T65-R was markedly later than T65-T under the two SD conditions. Consequently, T65-R was earlier in heading than T65-T under the LD conditions at the critical stage. They were similar in heading under the 13.5-h photoperiod condition and the summer-sowing PF condition in which the day lengths at the critical stage were about 13 h. T65-R was later than T65-T under the SD conditions.

### DTHs of T65wx and three late NILs under the six environmental conditions involving various day lengths

Under the PF condition in 2011, LF3, LF1 and LF2 were 33.2, 38.9 and 51.9 days later in heading, respectively, than T65wx (Table 5). The four lines were in the same order in 2018 as in 2011. Day lengths at the critical stage in 2011 and 2018, respectively, were 14:21 and 14:23, 13:45 and 13:34, 13:35 and 13:18, and 13:10 and 12:57, respectively, in T65wx, LF3, LF1 and LF2, which may be long, or intermediate for the four lines. LF3, LF1 and LF2 were 14.5 to 17.8 days longer in DTH in 2018 than in 2011, while T65wx was 3.9 days shorter in 2018. One cause of the longer DTHs in the three late NILs could be the earlier sowing in 2018 as mentioned above. However, we cannot explain the considerable between-year difference in each of the three late NILs. Nevertheless, mutual differences in DTH among the three late NILs in 2018 were similar to those in 2011: the between-year difference in each of the mutual differences was from 1.1 to 3.3 days. Consequently, under each of the two spring-sowing PF (LD) conditions, LF1 (*se-pat*) and LF3 (*Se1-pat*(t)) were later in heading than T65wx, and LF2 (*Se1-pat*(t), *se-pat*) was later than both of LF1 and LF3.

Under the 13.5-h photoperiod condition, LF3, LF2 and T65wx were in the same order as those in the two spring-sowing PF conditions, whereas LF1 was 6.9 days earlier than LF3.

Under the summer-sowing PF condition, LF2, LF3 and T65wx were similar to one another in heading although the difference between the latter two lines was significant; however, LF1 was 17.7 days later than T65wx. Day lengths at the critical stage were 13:04, 12:59, 12:28 and 13:02, respectively, in T65wx, LF3, LF1 and LF2, which were intermediate between SD and LD for them.

Under the 10-h photoperiod condition, LF3 was 5.3 days earlier in heading than T65wx. LF2 was 1.4 days earlier than T65wx although being not statistically significant. LF1 was 33.8 days later than T65wx.

Under the Nat-L growth chamber condition, in which day lengths at the critical stage were from 9:56 to 10:24 in the four lines, LF3 and LF2 were 16.3 and 15.2 days earlier in heading, respectively, than T65wx, and the difference between the former two lines was not significant. On the other hand, LF1 was 24.7 days later than T65wx.

Consequently, under each SD condition, LF2 (*Se1-pat*(t), *se-pat*) as well as LF3 (*Se1-pat*(t) was earlier in heading than T65wx, while LF1 (*se-pat*) was later than T65wx.

### Number of developed leaves, and lengths of culm and panicle in T65-R and its early NILs, and T65-T

Under the 10-h photoperiod condition, the order of number of developed leaves in T65-R, the early NILs, and T65-T were ER50 ≤ ER20 ≤ ER40 ≤ ER1 ≤ ER21 < T65-T < T65-R, and ER50 < ER21 (Table 6). In this trait, the five early lines were lower than T65-T, and T65-R was the highest. The delay of heading in T65-R was accompanied by an increase of the number of developed leaves. The order in this trait under the 13.5-h photoperiod condition was similar to that under the 10-h photoperiod condition, although ER21 was lower than ER1 and ER40, and T65R ≑ T65T. The correlation coefficients between DTH and number of developed leaves among the seven lines were 0.991 and 0.972 in the former and latter conditions, respectively, which are statistically significant at the 1% level: y = 12.8x – 54.7 and y = 16.4x – 102.5, respectively, where x = number of developed leaves and y = DTH. Consequently, there was a close relationship between the number of developed leaves and variation of DTH caused by *Ef1*, *Efx*, *m-Ef1* and *Ac-ef1*.

**Table 6:** Number of developed leaves, and lengths of culm and panicle in T65-R, T65wx and their early and late NILs

Line/ Variety	10-h photoperiod^a^	13.5-h photoperiod^a^	Spring-sowing paddy field in 2018	
	No. of leaves^b^	No. of leaves^c^	Culm length^d^	Panicle length^d^
ER50	9.2 ab	10.2 a	73.4 a	18.2 a
ER40	10.0 bc	11.4 bc	80.5 b	20.5 b
ER20	9.7 ab	10.7 ab	79.0 b	20.0 b
ER1	10.0 bc	11.9 cd	84.6 c	20.2 b
ER21	10.8 cd	10.9 b	78.4 b	20.8 b
T65-R	13.0 f	12.7 e	86.5 cd	22.9 cd
T65-T	12.0 e	12.6 de	80.2 b	22.6 cd
T65wx	11.0 d	12.9 e	78.2 b	22.3 c
LF3	9.0 a	14.1 f	97.0 e	23.3 cd
LF1	13.2 f	14.1 f	80.8 b	22.8 cd
LF2	9.5 ab	14.5 f	87.9 d	20.2 b

Values followed by the same letter within the same column are not statistically different at the 5% level, in accordance with Tukey-Kramer’s method (Sokal and Rohlf [28])

^a^Artificial light-type growth chamber

^b^5 to 7 plants per line were measured

^c^11 to 17 plants per line were measured

^d^19 to 24 plants per line were measured

Under the PF condition in 2018, the culm lengths of the early NILs, T65-R and T65-T were in the order of ER50 < ER21 ≤ ER20 ≤ ER40 ≤ T65-T < ER1 ≤ T65-R. ER50, ER21, ER20, ER40 and T65-T were significantly lower than T65-R, and ER50 was the lowest. However, ER1 was not significantly different from T65-R. The correlation coefficient between DTH and culm length among the seven lines was 0.664, which was not statistically significant at the 5% level. Hence, the decreases of DTH caused by *Efx* (ER21), the combination of *Efx* and *Ef1* (ER20), that of *Ef1* and *m-Ef1* (ER40), and all of the three alleles (ER50) brought about reduction in culm length. However, *Ef1* (ER1) alone did not reduce culm length. *Ac-ef1* may have increased culm length, which is in accordance with the result by Tsai [36]. The panicle lengths of ER50, ER20, ER1, ER40 and ER21 were significantly lower than T65-R, and ER50 was the lowest. However, T65-T was not significantly different from T65-R. The correlation coefficient between culm length and panicle length among the seven lines was 0.715, which was not statistically significant at the 5% level.

### Number of developed leaves, and lengths of culm and panicle in T65wx and three late NILs

Under the 10-h photoperiod condition, LF3 and LF2 were lower than T65wx in number of developed leaves but LF1 was higher than T65wx; and LF3 was similar to LF2 (Table 6). Under the 13.5-h photoperiod condition, the three late lines were significantly higher than T65wx, while there were not significant differences among the three late NILs. The correlation coefficients between DTH and number of developed leaves among the four lines were 0.934 and 0.969 in the former and latter conditions, respectively, which were statistically not significant and significant at the 5% level; y = 19.1x – 134.2 (y and x: see previous section) in the later environmental condition. Hence, the delay and acceleration of heading caused by *Se1-pat*(t) and delay by *se-pat* were closely accompanied by an increase and decrease of the number of developed leaves at least under the 13.5-h photoperiod condition.

Under the PF condition in 2018, T65wx and the late NILs were in the order of T65wx ≤ LF1 < LF2 < LF3 in culm length, and LF3 was the highest. In term of panicle length, LF3 and LF1 were not significantly different from T65wx, while LF2 was lower than T65wx.

## Discussion

The early NILs and T65-R were in similar orders of DTH under the three PF conditions, although their DTHs were 8.8 to 13.7 days shorter under the summer-sowing PF condition in 2014 than in the PF condition in 2018. We compared the DTHs of the five early NILs and T65-R in 2014 with those in a mid-August-sowing PF condition in Okinawa (Faculty of Agriculture, University of the Ryukus, 26°15′N) [9]: the correlation coefficient between the former and latter conditions is 0.941 which is statistically significant at the 1% level, and the average DTH of the six lines in the former was later by 3.1 days than in the latter. Hence, similar results were obtained under the condition of 13:04 to 13:44 day-length at the critical stage in 2014 (Table 3) and under the day length condition of about 12 h at the critical stage in Okinawa (during September and October, according to the database of National Astronomical Observatory of Japan), in which temperature was high in the latter condition. Consequently, *Ef1*, *Efx*, their combination, and the additional enhancing effect of *m-Ef1*^*b*^ controlled DTH constantly under the LD conditions, the day length conditions of 13:04 to 13:44 and about 12 h, all in the PF conditions. However, under each of the two SD conditions, ER40 (*e1* = *m-Ef1*, *Ef1*) was not significantly different from ER1 (*E1* = *M-Ef1*, *Ef1*), and similarly, ER50 (*e1* = *m-Ef1*, *Ef1*, *Efx*) was not significantly different from ER20 (*E1* = *M-Ef1*, *Ef1*, *Efx*) (Table 2 and Table 5), probably because *E1* did not increased DTH under the SD conditions. Results of Uwatoko et al. [38] suggest that in the coexistence of *Ef1*, *E1* increased DTH under a 14-h photoperiod condition, but *E1* did not increase DTH under a 10-h photoperiod condition, from their experiments by using a Art-L chamber set at 22 °C as well as at 28 °C. These results support our results for ER40 and ER1, and ER50 and ER20 under the SD conditions.

Tsai [35] reported that T65-R was 7.7 and 4.2 days earlier in heading, respectively, than T65-T in the first and second crop seasons of a year in Taichung, Taiwan, which does not contradict the results obtained in this study under the spring-sowing PF conditions (Table 5). Tsai [35] assumed that this varietal difference is due to the allelic difference between *Ac-ef1* and *ac-ef1* harbored in T65-R and T65-T, respectively, and the former allele affect *ef1* and accelerate heading; additionally that a differentiation of *ef1* of T65-T to *ef1*^R^(t) of T65-R is related to this difference, although it seems to have not been sufficiently verified. However, T65-R was about 19 and 10 days later in heading, respectively, than T65-T under the short-photoperiod conditions in the Art-L and Nat-L growth chambers (Tables 3 and 5). Furthermore, under a 8-h photoperiod condition in the same Art-L growth chamber, T65-R was 22.9 days later in heading than T65-T (Murai unpublished). It is difficult to presume that the allelic difference between T65-T and T65-R at the *Ef1* locus, which controls the BVG, was related to this difference regarding photo-sensitivity. Hence, it is inferred that *Ac-ef1* involves the effect of delaying heading under short-photoperiod conditions. This is a notably interesting genic effect of delaying heading under the short-photoperiod conditions, because it is known that photo-sensitivity alleles at the same and different loci, viz. *Se1-u* [44], *Se1-pat*(t) (Table 5), a dominant allele at the *Hd16* locus (Matsubara et al. [18]), and a functional allele at the *Hd2* locus [14], accelerate heading under short-photoperiod conditions. Nevertheless, the five NILs were earlier in heading than T65-R as well as T65-T under the two short-photoperiod conditions, even if it is postulated that they carry *Ac-ef1* (Tables 2 and 5). Tsai [35] indicated that *Ac-ef1* does not influence the effect of *Ef1* on accelerating heading, which supports the finding that the effect of *Ef1* for earliness in ER1, ER20, ER40 and ER50 were not prevented even under the SD conditions (Table 5). Regarding ER21, it is presumed that *Efx* masked the genic effect of *Ac-ef1* under the SD conditions, although further genetical experiments would be necessary to verify this presumption. We are not aware of any previous reports regarding such genes/alleles that delay heading under SD conditions in rice. However, it cannot be stated that this phenomenon is not due to a gene/allele at a locus different from the *Ac-ef1* locus. We have been conducting genetical research using the F_1_ between T65-R and T65-T and its descendants to clarify the genetic mechanism of this specific response to short photoperiods, and obtained results suggesting single-genic control for the phenomenon (Murai unpublished). In addition, mapping and cloning for *Ac-ef1* are necessary to clarify the intra-genic constitution and physiological function of the *Ac-ef1* locus at the molecular level.

LF3 (*Se1-pat*(t)) delayed heading from T65wx by 33.2 and 48.1 days, respectively, under the two spring-sowing PF conditions, probably because LF3 was not able to prepare to initiate panicle development until day length decreased to its threshold day lengths of 13:45 and 13:34, respectively, on 4th August 2011 and 10th August 2018 (Tables 3 and 5). LF3 was similar to T65wx in DTH under the summer-sowing PF condition, probably because high temperatures during July and August accelerated growth to attain sufficient the BVG (Table 4), and the shoot apices of LF3 prepared to change into panicle primordia in a day length shorter than its threshold (12:59 at the critical stage of 29th August in 2014). On the other hand, LF3 was 5.3 and 16.3 days earlier in heading, respectively, than T65wx under the SD conditions in the Art-L and Nat-L growth chambers. Nevertheless, we cannot explain the higher difference in the latter condition. LF3 was 27.8 days later in heading than T65wx under an early-May-sowing PF condition at the National Institute of Genetics (35°7′N), Mishima, Kanagawa, Japan [9]. Yokoo and Kikuchi [44] developed a pair of NILs carrying *Se1-u* and *Se1-e* in the genetic background of Fujusaka 5, a representative variety of northern Japan. The former line was 30 and 34 days later in heading than the latter line under spring-sowing conditions in 2015 and 2016, respectively, in the same PF as in the present study (Murai unpublished). Therefore, *Se1-pat*(t) could be an allele identical to *Se1-u* or its allele with a similar effect, both of which originate from the respective *indica* varieties.

LF1 (*se-pat*) was later in heading by 17.2 to 57.1 days than T65wx under the three PF conditions and the three growth chamber conditions, regardless of day length (Tables 3 and 5). LF1 was 17.0 and 20.8 days later in heading than T65wx, respectively, under an early-May-sowing PF condition in Mishima and a late-May-sowing greenhouse condition at the Research Faculty of Agriculture, Hokkaido University (42°55′N), Sapporo, Hokkaido, Japan [9]. In addition, Hagiwara et al. [6] reported that *se-pat* increased DTH under a SD condition. Hence, it could be concluded that *se-pat* involves the effect of elongating the BVG.

The *se-pat* elongated DTH by adding its effect over that of *Se1-pat*(t) in LF2 under the 13.5-h photoperiod condition and the two spring-sowing PF conditions in which day lengths at the critical stage were 13:10 and 12:57, respectively, in 2011 and 2018 (Table 3). Day lengths at the 55 days before heading in LF2 were 13:46 and 13:34, respectively, in 2011 and 2018 (the database of the National Astronomical Observatory of Japan), which were almost identical to the day lengths at the critical stage (35 days before heading) in LF3 in the corresponding years (Table 3). Before this time (55 days before heading) in each year, day length seems to have been sufficiently long to prevent the preparation for panicle development by the effect of *Se1-pat*(t) on photosensitivity. In LF2, it is inferred that *se-pat* increased DTH when *Se1-pat*(t) exerted its effect for elongating DTH. Similar results were obtained at Mishima [9] and Sapporo [6]. Under the two SD conditions, however, this effect of *se-pat* was almost masked when it coexisted with *Se1-pat*(t) in LF2, because LF2 was similar to LF3 in heading under either of the SD conditions. Results obtained by Uwatoko et al. [38] suggest that in the coexistence of *Se1-pat*(t), *ef1* increased DTH not only under a 13-h photoperiod condition but also under a 10-h photoperiod condition, in the common genetic background of T65wx, indicating that the effect of *ef1* on increasing the BVG was not masked, being unlike the case of LF2 and LF3 under the SD conditions (Table 5). As far as we know, there are not previous reports that the effect of a gene on increasing the BVG is masked by the coexistence of a functional allele at the *Se1* locus under a short day condition.

*Hd3a* and *RFT1* (*Rice Flowering Locus T1*) encode mobile flowering signals that are promising candidates for florigen in rice [12], which are adjacently located on the short arm of chromosome 6. *Hd3a* and *RFT1* are major floral activators under SD and LD conditions, respectively [13]. *Se1* (*Hd1*) involves a zinc finger domain that encodes a zinc finger protein [42]. *Hd1* suppresses the activity of *Hd3a* under LD conditions resulting in delay of heading [16], whereas it up-regulates the activity of *Hd3a* under SD conditions, accelerating heading [11]. Hagiwara et al. [6] suggested that *se-pat* is an allele at the *RFT1* locus, which is caused by an amino acid substitution from *se-pat*^*+*^ to *se-pat*. Ogiso-Tanaka et al. [20] determined that the amino acid substitution at the *RFT1* locus is the cause of a functional defect in *RFT1*, which was found in ‘Nona Bokra’ and other *indica* varieties. According to Hagiwara et al. [6] and Ogiso-Tanaka et al. [20], under LD conditions, *se-pat* or the allele involving the same amino acid substitution at the *RFT1* locus increased DTH, which supports the results regarding LF1 in the present study (Table 5). Under the two SD conditions (Table 5), *Se1-pat*(t) accelerated heading, and it almost completely masked the effect of delaying heading by *se-pat*. It is inferred that under the SD conditions, *Se1-pat*(t) activated *Hd3a* resulting in a level of florigen activity higher in LF2 than in T65wx, even though *se-pat* did not contribute to produce florigen.

Kuriyama [15] reported that difference in the number of developed leaves among the early and late rice varieties, and decrease of DTH by each of the SD treatments was accompanied by a decrease in the number of developed leaves in each of the photosensitive varieties examined. The results of the present study indicate that the acceleration of heading by *Se1-pat*(t) observed from the 13.5-h photoperiod to the 10-h photoperiod conditions was accompanied by a decrease in the number of developed leaves in each of LF3 and LF2 (Tables 5 and 6), which is in accordance with the results of Yokoo et al. [47]. The delay of heading caused by *se-pat* from T65wx to LF1 was accompanied by an increase of number of developed leaves in each of the SD and the 13.5-h photoperiod conditions. The delays of heading by *Se1-pat*(t) and the combined effect of *Se1-pat*(t) and *se-pat* from T65wx to LF3 and LF2 were accompanied by an increases in the number of developed leaves in the 13.5-h photoperiod condition. The accelerations of heading caused by *Ef1* and *Efx*, their combined effect and the additional effect of the enhancer were accompanied by decreases of number of developed leaves. Therefore, the changes of DTH caused by the earliness and the lateness genes could be principally due to the changes in the number of developed leaves to flag leaf rather than changes in the developing speed of leaves.

Yokoo et al. [47] indicated that *Se1-u* increases culm length, which is in accordance with the result regarding the *Se1-pat*(t) obtained by using LF3 and T65wx. On the other hand, *Efx*, the combination of *Efx* and *Ef1*, that of *Ef1* and *m-Ef1*, and all of the three alleles reduced culm length; particularly, ER50 was the lowest. Reduction in panicle length was noticed in all of the five early NILs. According to Tsai and Oka [31], *Ef1* pleiotropically reduces plant height, panicle length and grain number per panicle, resulting in lower yield. There is a possibility that decrease in panicle length caused by the earliness genes brings about lower yield. However, Bozu 5, which harbors *Ef1* and *m-Ef1* like ER40, had been a representative cultivar in Hokkaido around 100 years ago. It would be useful to examine whether the two earliness alleles, the enhancer and their combination mentioned above exert any effect on yield or not. To answer this question, yield tests for the five early NILs and T65-R should be conducted.

As far as we know, there have been no previous reports regarding the specific response of *Ac-ef1* in heading. In addition, it was found that the effect of *se-pat* allele for longer the BVG was almost completely masked by the effect of the functional allele at the *Se1* locus under the SD conditions, by using the NILs and the recurrent parent. Even if there are many reports regarding genes controlling heading time, unknown genic effects and genic interactions concerning heading may still remain to be clarified in the genetic diversity of rice.

## Data Availability

The original datasets used and analyzed during the current study are available from the corresponding author on reasonable request.

## Abbreviations

Art-L: Artificial light
BVG: Basic vegetative phase
DTH: Days to heading
LD: Long day
Nat-L: Natural light
NILs: Nearly isogenic lines
PF: Paddy field
SD: Short day
